# Using Wearable Inertial Sensors to Assess Mobility of Patients With Hematologic Cancer and Associations With Chemotherapy-Related Symptoms Before Autologous Hematopoietic Stem Cell Transplant: Cross-sectional Study

**DOI:** 10.2196/39271

**Published:** 2022-12-08

**Authors:** Meghan B Skiba, Graham Harker, Carolyn Guidarelli, Mahmoud El-Gohary, Fay Horak, Eric J Roeland, Rebecca Silbermann, Brandon Hayes-Lattin, Kerri Winters-Stone

**Affiliations:** 1 Biobehavioral Health Science Division College of Nursing University of Arizona Tucson, AZ United States; 2 The University of Arizona Cancer Center University of Arizona Tucson, AZ United States; 3 Department of Neurology School of Medicine Oregon Health & Science University Portland, OR United States; 4 Division of Oncological Sciences Knight Cancer Institute Oregon Health & Science University Portland, OR United States; 5 APDM, Inc, a division of Clario International Portland, OR United States; 6 Division of Hematology and Medical Oncology School of Medicine Oregon Health & Science University Portland, OR United States

**Keywords:** wearable inertial sensor, mobility, gait, induction chemotherapy, autologous hematopoietic stem cell transplant, autoHSCT, chemotherapy-related symptoms

## Abstract

**Background:**

Wearable sensors could be a simple way to quantify and characterize mobility in patients with hematologic cancer scheduled to receive autologous hematopoietic stem cell transplant (autoHSCT) and how they may be related to common treatment-related symptoms and side effects of induction chemotherapy.

**Objective:**

We aimed to conduct a cross-sectional study comparing mobility in patients scheduled to receive autoHSCT with that in healthy, age-matched adult controls and determine the relationships between patient mobility and chemotherapy-related symptoms.

**Methods:**

Patients scheduled to receive autoHSCT (78/156, 50%) and controls (78/156, 50%) completed the prescribed performance tests using wearable inertial sensors to quantify mobility including turning (turn duration and number of steps), gait (gait speed, stride time, stride time variability, double support time, coronal trunk range of motion, heel strike angle, and distance traveled), and balance (coronal sway, coronal range, coronal velocity, coronal centroidal frequency, sagittal sway, sagittal range, sagittal velocity, and sagittal centroidal frequency). Patients completed the validated patient-reported questionnaires to assess symptoms common to chemotherapy: chemotherapy-induced peripheral neuropathy (Functional Assessment of Cancer Therapy/Gynecologic Oncology Group–Neurotoxicity subscale), nausea and pain (European Organization for Research and Treatment of Cancer Quality of Life Questionnaire), fatigue (Patient-Reported Outcomes Measurement Information System Fatigue Short Form 8a), vertigo (Vertigo Symptom Scale–short form), and depression (Center for Epidemiological Studies–Depression). Paired, 2-sided *t* tests were used to compare mobility between patients and controls. Stepwise multivariable linear regression models were used to evaluate associations between patient mobility and symptoms.

**Results:**

Patients aged 60.3 (SD 10.3) years had significantly worse turning (turn duration; *P*<.001), gait (gait speed, stride time, stride time variability, double support time, heel strike angle, stride length, and distance traveled; all *P*<.001), and balance (coronal sway; *P*<.001, range; *P*<.001, velocity; *P*=.02, and frequency; *P*=.02; and sagittal range; *P*=.008) than controls. In patients, high nausea was associated with worse stride time variability (ß=.001; *P*=.005) and heel strike angle (ß=−.088; *P*=.02). Pain was associated with worse gait speed (ß=−.003; *P*=.003), stride time variability (ß=.012; *P*=.02), stride length (ß=−.002; *P*=.004), and distance traveled (ß=−.786; *P*=.005). Nausea and pain explained 17% to 33% and 14% to 36% of gait variance measured in patients, respectively.

**Conclusions:**

Patients scheduled to receive autoHSCT demonstrated worse mobility in multiple turning, gait, and balance domains compared with controls, potentially related in part to nausea and pain. Wearable inertial sensors used in the clinic setting could provide granular information about mobility before further treatment, which may in turn benefit from rehabilitation or symptom management. Future longitudinal studies are needed to better understand temporal changes in mobility and symptoms across the treatment trajectory to optimally time, design, and implement strategies, to preserve functioning in patients with hematologic cancer in the long term.

## Introduction

### Background

The increasing frequency of autologous hematopoietic stem cell transplant (autoHSCT) to treat hematologic malignancies, especially among older adults, has contributed to increased survival [1,2]. AutoHSCT is preceded by myeloablative induction chemotherapy [3], which often leads to deconditioning and worsening of symptoms before transplant [4,5]. These pretransplant treatment-related impacts could predispose patients to altered mobility (ie, altered gait and balance) that could worsen after transplant and threaten patient’s functioning and quality of life [6,7]. Mobility declines have broad health implications, as patients undergoing transplants who report low physical functioning are at high risk for morbidity and mortality following transplant [8,9]. Over the past few years, a few studies have evaluated the feasibility and potential clinical utility of wearable sensors in the oncology setting [10-12]. Wearable sensors could describe specific patterns of mobility impairment and their potential attribution to treatment-related symptoms and potentially identify patient risk at discrete intervals along the treatment trajectory. In turn, this information could be used to inform timing and design of rehabilitation and symptom management strategies to positively affect clinical outcomes for the patient with hematologic cancer [13].

Before transplant, patients undergo conditioning therapy, which can include any combination of radiation therapy, immunotherapy, or induction chemotherapy [3], all of which cause treatment-related symptoms and side effects that may linger into transplant [14]. Induction chemotherapy, in particular, can cause symptoms known to affect mobility including fatigue, neuropathy, vestibular dysfunction, dizziness, and pain [15]. Symptom clusters in patients undergoing transplant include fatigue, weakness, and anorexia; anxiety and depression; and nausea and vomiting [16]. These symptom clusters are associated with decreased self-reported physical functioning during autoHSCT and increased fall risk [17,18]. Current knowledge has relied on patient-reported measures of physical functioning, which can be less sensitive and informative and more prone to bias than objective measures of mobility and functioning [19,20]. It is also possible that using self-report may underestimate the degree of functional limitation among patients before autoHSCT. In addition, identifying the potential influence of treatment-related symptoms that are present at the time of transplant on mobility could identify patients at high risk for further decline after autoHSCT and who could benefit from appropriately timed rehabilitation and symptom management.

Objective mobility measurements can assess turning, gait, and balance during prescribed tasks, such as walking at a usual pace, walking while turning, and standing in place. Using technology to capture mobility measures can provide greater precision, sensitivity, and granularity of information than clinical or field tests [21-23]. Characterizing the mobility characteristics of turning, gait, and balance using indices of support, stance, swing, spatial temporal patterns, stability, and range of motion typically requires advanced laboratory techniques (ie, motion cameras) that limit their application in nonresearch settings. Advancements in wearable sensors to quantify the same laboratory-based assessments in a clinic or home setting widen the scope of objective mobility assessment to include clinical populations undergoing intensive treatment and requiring hospitalization, such as patients undergoing autoHSCT. So far, a single study using insole sensors to measure gait patterns in patients after receiving allogeneic hematopoietic stem cell transplant (HSCT) reported slower walking speeds and shorter stride times than healthy matched controls, suggesting that treatment may have altered gait [24]. However, as gait was measured after treatment, it remains unknown whether patients already experienced some mobility limitations from treatments before transplant and whether and which persistent symptoms may be associated with mobility in patients receiving autoHSCT.

### Objectives

We conducted a cross-sectional study using wearable inertial sensors to measure mobility in patients with hematologic cancer after induction chemotherapy and before autoHSCT to identify (1) differences in mobility between patients and age-matched controls and (2) whether and which symptoms typically related to chemotherapy may be associated with pretransplant mobility in patients.

## Methods

### Study Design

We used a case-control design to compare the mobility of 78 patients with hematologic cancer before transplant with that of healthy age-matched controls and a cross-sectional design to identify chemotherapy-related symptoms associated with mobility in patients.

### Participants and Setting

Eligible patients were recruited through the Oregon Health & Science University Knight Cancer Institute Center for Hematologic Malignancies HSCT unit. Eligible patients were those who were scheduled to receive autoHSCT for a hematopoietic or lymphatic malignancy, were aged ≥21 years at the time of enrollment, had no cognitive difficulties that precluded completing surveys, were participating in performance testing, provided informed consent, and had no preexisting medical conditions that significantly affect mobility (ie, severe dystrophy, severe spasticity, epilepsy, seizures, Alzheimer disease, dementia, severe balance disorder, and inability to ambulate independently). Patients completed assessments after the completion of initial induction chemotherapy and within 2 weeks before hospitalization for autoHSCT.

### Age-Matched Controls

Age-matched controls were selected from a preexisting sample of healthy adults recruited from the local community for 2 study protocols [25,26]. Eligible controls had no history of falls, chronic diseases including cancer, significant neurological or musculoskeletal impairment, or medication use that affects mobility or limits their ability to follow instructions or provide informed consent. Controls were age-matched to participants according to age at the time of assessment within 1 year.

### Ethics Approval

The Oregon Health & Science University institutional review board approved the study (16760), and informed consent was obtained from all participants before data collection. Participant data were deidentified using individual code numbers assigned upon enrollment. Participants were not compensated for participating in the study. The survey and mobility assessment took approximately 30 to 45 minutes to complete and thus was not considered to pose a significant burden to participants.

### Demographic Measurements

Patient demographics (age, sex, ethnicity or race, education, marital status, employment, and history of falls in the previous year) were self-reported. Comorbidities were determined using the Functional Comorbidity Index, a self-administered 18-item checklist of chronic conditions that affect physical functioning [27]. Self-reported cancer diagnosis and treatment history were adjudicated by the research staff. Height and weight were measured in the clinic, and BMI was calculated as kg/m^2^. The control group’s self-reported demographic data included age, sex, height, weight, health history, and education.

### Objective Mobility Assessment

**Figure 1:**
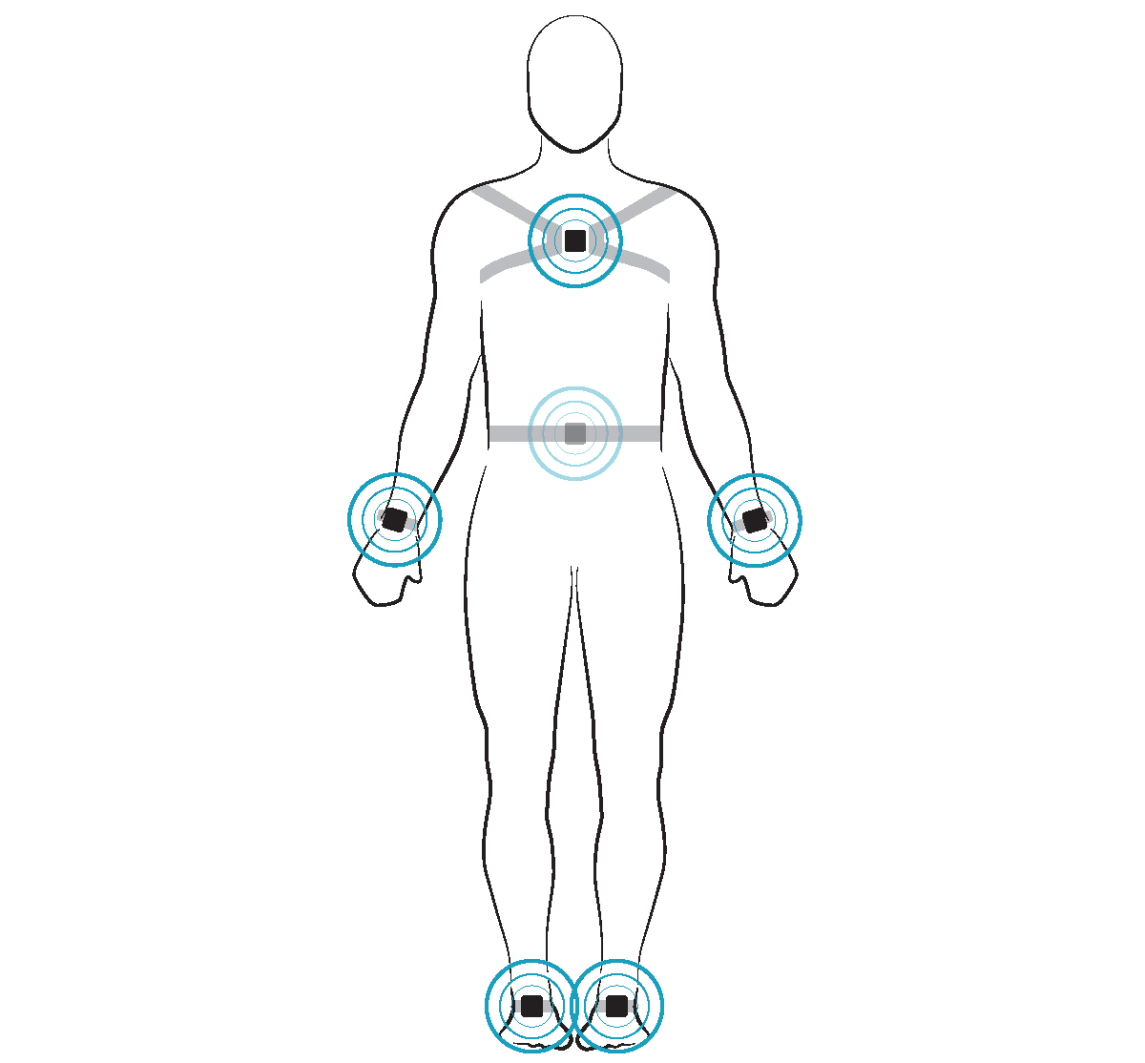
Inertial sensor placement (Mobility Lab Opal; APDM, Inc). In total, 6 sensors are placed—sternum (1 sensor; centered just below the collar bones, on the flat part of the chest), lumbar spine (1 sensor; centered at the base of the spine), wrist (2 sensors; on the wrist, similar to a watch), and ankles (2 sensors; centered on the front of the ankle). The figure was reproduced with permission from APDM, Inc.

Objective mobility measures were assessed using Mobility Lab (APDM, Inc), a portable system of unobtrusive, body-worn, wireless, inertial sensors that quickly and automatically provide objective mobility measures, including turning, gait, and balance [28-30]. Patients’ Mobility Lab assessments were collected in the clinic using available space (eg, hallways) during a single appointment. Participants wore inertial sensors (Opal; APDM, Inc), placed at the sternum, lumbar spine, wrists, and ankles (Figure 1), and performed 2 standard physical functioning assessments—a 6-minute walk test (6MWT) and a 30-second quiet stance [19]. The 6MWT assesses distance walked over 6 minutes and is one of the most established outcome measures of functional mobility in clinical trials [31,32]. Participants walked at their usual pace for 6 minutes on a 20-meter course. Each full lap provided gait and turns averaged together, considerably reducing variability and performance bias compared with a single walk [33,34]. For controls, if a 6MWT was not performed owing to differences in protocol at the time of consent, a 400-meter walk was completed [25,26], which provides similar estimates of turning and gait [35]. Balance was measured using a 30-second quiet stance test, where participants stood as still as possible for 30 seconds with eyes open, feet together, and hands on their hips. Measures specific to turning, gait, and balance selected for these analyses (Table 1) have been previously used to assess fall risk, including dynamics during turning, postural adjustments associated with step initiation, spatial and temporal components of gait, and postural sway during standing balance [25,36-38]. Data processing was performed using Mobility Lab (version 2; APDM, Inc) and established algorithms [28,39]. The algorithms account for difference in physical stature (eg, height) of participants and in physical functioning assessment protocols for samples recruited at different times, allowing for a large sample of community-dwelling healthy adults with valid mobility data to select age-matched controls.

**Table 1:** Definitions of selected Mobility Lab mobility measures of turning, gait, and balance.

Measure			Definition
**Turning (6MWT^a^)**			
	Turn duration (s)	Duration of 180° turn	
	Number of steps	Number of steps during 180° turn	
**Gait (6MWT)**			
	Gait speed (m/s)	Forward speed of the individual, measured as the forward distance traveled during the gait cycle divided by the gait cycle duration	
	Stride time (s)	Duration of a full gait cycle, measured from the left foot’s initial contact to the next initial contact of the left foot	
	Stride time variability (%)	Coefficient of variation stride length (SD/mean)	
	Double support time (%)	Rate of gait cycle while both feet are on the ground	
	Coronal trunk ROM^b^ (°)	Angular range of the lumbar spine in the coronal plane	
	Heel strike angle (°)	Angle of the foot at the point of initial contact; the pitch of the foot is 0 when flat and positive when the heel contacts first	
	Stride length (m)	Forward distance traveled by a foot during a gait cycle	
	Distance (m)	Total distance traveled during the timed test, at usual walking speed	
**Balance (30-second quiet stance)**			
	Coronal sway RMS^c^ (m/s^2^)	Amplitude of lateral sway	
	Coronal range (m/s^2^)	Angular range of the lateral thoracic spine (roll)	
	Coronal velocity (m/s)	Mean velocity of lateral sway	
	Coronal centroidal frequency (Hz)	Frequency of centroidal lateral sway	
	Sagittal sway RMS (m/s^2^)	Amplitude of anterior-posterior sway	
	Sagittal range (m/s^2^)	Angular range of the anterior-posterior thoracic spine (pitch)	
	Sagittal velocity (m/s)	Mean velocity of anterior-posterior sway	
	Sagittal centroidal frequency (Hz)	Frequency of centroidal anterior-posterior sway	

^a^6MWT: 6-minute walk test.

^b^ROM: range of motion.

^c^RMS: root mean square.

### Patient-Reported Symptoms

#### Overview

For patients scheduled for autoHSCT, validated, patient-reported outcomes on symptoms typically associated with chemotherapy were collected using the following instruments and administered electronically in REDCap (Research Electronic Data Capture; Vanderbilt University) [40]. High scores indicate high level of symptoms for all questionnaires, unless otherwise described. Symptoms were not assessed for controls.

#### Chemotherapy-Induced Peripheral Neuropathy

Numbness, tingling, or uncomfortable sensations in hands and feet over the previous 7 days were measured using the 4-item Functional Assessment of Cancer Therapy/Gynecologic Oncology Group–Neurotoxicity subscale, a reliable and valid measure of chemotherapy-induced peripheral neuropathy (score range 0-16, where high scores indicate less-severe chemotherapy-induced peripheral neuropathy; minimally clinically important difference [MCID] 1.38-3.68) [41].

#### Nausea and Pain

Symptoms during the previous week were assessed using the European Organization for Research and Treatment of Cancer Quality of Life Questionnaire–nausea or vomiting and pain symptom subscales (score range 0-100; MCID 2.4-15.5 [nausea] and 14.4-28.5 [pain]) [42,43]. This questionnaire is an acceptable measure of chronic pain [44].

#### Fatigue

Fatigue over the previous week was determined using the Patient-Reported Outcomes Measurement Information System Fatigue Short Form 8a (score range 0-100; MCID 3-5) [45,46].

#### Vertigo

The Vertigo Symptom Scale–short form was used to measure vertigo, dizziness, and somatic anxiety over the past month (score range 0-60; MCID 3) [47,48].

#### Depression

Depressive symptoms over the past week were assessed using the Center for Epidemiological Studies–Depression scale (score range 0-16; MCID 9-11) [49,50].

### Statistical Analysis

Distributions were inspected for normality; balance measures were log transformed to improve normality, but model results were consistent; therefore, nontransformed variables and parametric tests were used for all analyses. Demographic characteristics were assessed using descriptive statistics, and paired, 2-sided *t* tests were used to determine the differences in mobility between patients and matched controls.

Linear regression models were used to determine the association between symptoms and mobility. Univariate linear regression models with α≤.05 were used to determine the model demographic control variables. The final models were adjusted for age, sex, and BMI. Symptom selection criteria for linear regression models were determined using Pearson correlations to mobility characteristics, with cutoff points ρ≥0.3 and α≤.10 [51]. Final stepwise multivariable linear regression models were built using α≤.05 with any mobility characteristic to symptoms and were externally validated with 1000 bootstrap replications. Variability of symptoms in mobility characteristics was estimated using standardized ß coefficients. Post hoc Benjamini-Hochberg false discovery rate adjustment [52] with α=.05 was completed for paired *t* tests and linear regression models. Analyses were completed using STATA (version 16.1; StataCorp, LLC), with α≤.05 for statistical significance.

## Results

### Participants

Between August 2017 and May 2019, 78 patients completed the Mobility Lab assessments before autoHSCT. The average age of patients before transplant was 60.3 (SD 10.3; range 31-76) years, and the most common cancer diagnosis was multiple myeloma (Table 2). The mean time since diagnosis to the scheduled autoHSCT was 9.9 (SD 11) months. All patients (78/78, 100) received induction chemotherapy before autoHSCT, with the average induction chemotherapy regimen lasting 4.7 (SD 3.2) months. In the year before the transplant, 17% (13/78) of the patients experienced a fall. The average age of matched controls (78/156, 50%) was 60.2 (SD 10.4) years. Most patients were men (50/78, 64%), whereas most controls were women (57/78, 73%). Controls had lower BMI and attained higher level of education than patients, and the control group had a high proportion of women compared with the patient group.

**Table 2:** Demographics and clinical characteristics of patients with hematologic cancer scheduled for autoHSCT^a^ compared with that of healthy, age-matched controls (N=156).

Characteristics		Patients scheduled for autoHSCT (n=78, 50%)	Healthy controls (n=78, 50%)
Age (years), mean (SD)		60.3 (10.3)	60.2 (10.4)
**Sex, n (%)**			
	Female	28 (36)	57 (73)
	Male	50 (64)	21 (27)
**Ethnicity, n (%)**			
	Non-Hispanic	69 (88)	N/A^b^
	Declined to answer	9 (12)	N/A
**Race, n (%)**			
	White	63 (81)	N/A
	Non-White^c^	5 (6)	N/A
	Declined to answer	10 (13)	N/A
**Education, n (%^d^)**			
	High school diploma or equivalent	20 (26)	2 (3)
	Some college or associate degree	18 (23)	8 (10)
	Bachelor’s degree or higher	31 (40)	68 (87)
	Declined to answer	9 (12)	N/A
**Marital status, n (%)**			
	Married or living with partner	53 (68)	N/A
	Divorced or separated	7 (9)	N/A
	Single	11 (14)	N/A
	Declined to answer	7 (9)	N/A
**Employment, n (%)**			
	Full time	23 (29)	N/A
	Part time	6 (8)	N/A
	Not working^e^	39 (50)	N/A
	Declined to answer	10 (13)	N/A
BMI (kg/m^2^), mean (SD)		29.8 (5.7)	25.2 (3.5)
Height (m), mean (SD)		1.7 (0.1)	1.6 (0.1)
Weight (kg), mean (SD)		88.9 (21.2)	66.3 (15.1)
**Cancer diagnosis, n (%)**			
	Multiple myeloma	53 (68)	N/A
	Hodgkin lymphoma	6 (8)	N/A
	Non-Hodgkin lymphoma	19 (24)	N/A
**Cancer stage^f^, n (%)**			
	I	11 (14)	N/A
	II	21 (27)	N/A
	III	21 (27)	N/A
	IV	12 (15)	N/A
	Missing or unknown	13 (17)	N/A
Time since diagnosis (months), mean (SD)		9.9 (11)	N/A
Received induction chemotherapy, n (%)		78 (100)	N/A
Duration of induction chemotherapy (months), mean (SD)		4.7 (3.2)	N/A
Time since last induction chemotherapy (days), mean (SD)		20.2 (91.9)	N/A
Received radiation treatment, n (%)		14 (18)	N/A
Functional Comorbidity Index score^g^, mean (SD)		1.3 (1.3)	N/A
History of fall in past year, n (%)		13 (17)	N/A

^a^autoHSCT: autologous hematopoietic stem cell transplant.

^b^N/A: not available; data were not collected for controls.

^c^Collapsed category including individuals who self-reported as being Asian, Black, or American Indian or Alaska Native or having >1 race.

^d^Percentages may not add up to 100% owing to rounding.

^e^Collapsed category including individuals who self-reported as being retired, unemployed, or homemaker. Disability status was not captured.

^f^Staging for multiple myeloma included International Staging System, Revised International Staging System, and Durie-Salmon staging classifications.

^g^Missing data <10%.

### Objective Mobility

Mobility was significantly worse across most measures among patients than among controls (Table 3). In the 6MWT, turn duration was 0.28 (SD 0.54) seconds longer for patients than for controls (*P*<.001). Patients demonstrated an altered gait pattern, as exhibited by significantly slower gait speed (mean −0.32, SD 0.25 seconds), longer stride time (mean 0.13, SD 0.13 seconds), higher stride time variability (mean 1.07%, SD 1.42%), longer double support time (mean 5.91%, SD 4.23%), shallower heel strike angle (mean 0.81°, SD 3.56°), shorter stride length (mean −0.18, SD 0.19 m), and shorter distance traveled (mean −60.01, SD 93.49 m) than controls (*P*<.001). During standing balance, patients had significantly larger coronal sway (mean 0.02, SD 0.03 m/s^2^; *P*<.001), longer coronal range (mean 0.10, SD 0.16 m/s^2^; *P*<.001), higher coronal velocity (mean 0.03, SD 0.10 m/s; *P*=.02), lower coronal centroidal frequency (mean −0.11, SD 0.39 Hz; *P*=.02), and longer sagittal range (mean 0.08, SD 0.27 m/s^2^; *P*=.008) than controls. Sensitivity analyses restricting the analytical sample to controls with gait data from the 6MWT (31/78, 40%) or who were both age-matched and sex-matched (44/78, 56%) yielded results consistent with those obtained using the full sample of controls.

**Table 3:** Comparison of mobility measures of turning, gait, and balance between patients with hematologic cancer scheduled for autoHSCT^a^ and age-matched healthy controls.

Measures		Patients scheduled for autoHSCT, mean (SD)	Healthy controls, mean (SD)	Difference, mean (SD)	*P* value^b^
**Turning**					
	Turn duration (s)	2.43 (0.37)	2.15 (0.40)	0.28 (0.54)	<.001
	Number of steps	4.04 (0.68)	4.07 (0.78)	−0.03 (0.97)	.82
**Gait**					
	Gait speed (m/s)	1.11 (0.19)	1.43 (0.15)	−0.32 (0.25)	<.001
	Stride time (s)	1.14 (0.11)	1 (0.08)	0.13 (0.13)	<.001
	Stride time variability (%)	3.55 (1.25)	2.48 (0.71)	1.07 (1.42)	<.001
	Double support time (%)	23.24 (3.62)	17.33 (2.99)	5.91 (4.23)	<.001
	Coronal trunk ROM^c^ (°)	7.14 (2.68)	6.33 (2.66)	0.81 (3.56)	.06
	Heel strike angle (°)	23 (5.57)	26.32 (4.23)	−3.32 (6.47)	<.001
	Stride length (m)	1.25 (0.16)	1.43 (0.13)	−0.18 (0.19)	<.001
	Distance (m)	375.76 (64)	435.78 (55.91)	−60.01 (93.49)	<.001
**Balance**					
	Coronal sway RMS^d^ (m/s^2^)	0.06 (0.02)	0.04 (0.02)	0.02 (0.03)	<.001
	Coronal range (m/s^2^)	0.33 (0.13)	0.23 (0.09)	0.10 (0.16)	<.001
	Coronal velocity (m/s)	0.10 (0.06)	0.07 (0.07)	0.03 (0.10)	.02
	Coronal centroidal frequency (Hz)	1.05 (0.32)	1.16 (0.26)	−0.11 (0.39)	.02
	Sagittal sway RMS (m/s^2^)	0.08 (0.04)	0.07 (0.04)	0.01 (0.05)	.06
	Sagittal range (m/s^2^)	0.43 (0.22)	0.34 (0.16)	0.08 (0.27)	.008
	Sagittal velocity (m/s)	0.15 (0.08)	0.13 (0.14)	0.02 (0.15)	.26
	Sagittal centroidal frequency (Hz)	0.95 (0.22)	0.96 (0.24)	−0.01 (0.32)	.83

^a^autoHSCT: autologous hematopoietic stem cell transplant.

^b^Paired, 2-sided *t* test, with Benjamini-Hochberg false discovery rate adjustment set at α=.05, and all significant *P* values remained significant.

^c^ROM: range of motion.

^d^RMS: root mean square.

### Mobility and Chemotherapy-Related Symptoms

Of the 78 patients with mobility data, 69 (88%) completed the patient-reported chemotherapy-related symptom questionnaires (Table 4). Reasons for missing questionnaires included incomplete responses, refusal, or acute illness. Patients with missing symptom data did not significantly differ from those with complete data on age (*P*=.73), BMI (*P*=.97), sex (*P*=.57), or Functional Comorbidity Index (*P*=.91); therefore, complete case analysis was conducted. Models were built for symptoms associations with gait only, because prespecified criteria for building regression models were met for symptoms and gait but not for turning or balance measurements. Symptoms that remained significantly associated with any gait metric were nausea and pain (Table 5). High nausea was associated with great stride time variability (ß=.023, 95% CI −0.007 to 0.039) and shallow heel strike angle (ß=−.088, 95% CI −0.160 to −0.017). High pain was associated with slow gait speed (ß=−.003, 95% CI −0.004 to –0.001), short stride length (ß=−.002, 95% CI −0.003 to −0.001), short distance (ß=−.786, 95% CI −1.321 to −0.252), and great stride time variability (ß=.012, 95% CI −0.002 to −0.023). Nausea better explained the variance in stride time variability (33%) and heel strike angle (31%), whereas pain better explained the variance in gait speed (36%), stride length (35%), and distance (34%).

**Table 4:** Chemotherapy-related symptom intensity among patients with hematologic cancer scheduled for autoHSCT^a^ (n=69).

Chemotherapy-related symptom	Measure possible score, range	Measure MCID^b^, range	Sample score, mean (SD)	Sample score, range
CIPN^c^	0-16	1.38-3.68	13.01 (3.63)	0-16
Nausea	0-100	2.4-15.5	11.35 (18.63)	0-100
Pain	0-100	14.4-28.5	27.05 (29.02)	0-100
Fatigue	0-100	3-5	53 (7.84)	33.1-69.8
Vertigo	0-60	3	4.81 (5.74)	0-31
Depression	0-60	9-11	9.19 (7.81)	0-39

^a^autoHSCT: autologous hematopoietic stem cell transplant.

^b^MCID: minimally clinically important difference.

^c^CIPN: chemotherapy-induced peripheral neuropathy.

**Table 5:** Associations between chemotherapy-related symptoms and gait characteristics among patients with hematologic cancer scheduled for autoHSCT^a^ (n=69).

Gait characteristics	Nausea				Pain		
	ß coefficient (95% CI)	Standardized ß coefficient	*P* value^b^	ß coefficient (95% CI)		Standardized ß coefficient	*P* value^b^
Gait speed (m/s)	−.002 (−0.005 to 0.0003)	−.189	.09	−.003 (−0.004 to −0.001)		−.355	.003
Stride time (s)	.001 (−0.001 to 0.002)	.127	.28	.001 (−0.0001 to 0.002)		.221	.07
Stride time variability (%)	.023 (−0.007 to 0.039)	.331	.005	.012 (−0.002 to 0.023)		.275	.02
Double support time (%)	.018 (−0.028 to 0.064)	.09	.43	.014 (−0.017 to 0.044)		.104	.38
Coronal trunk ROM^c^ (°)	.027 (−0.011 to 0.066)	.165	.16	−.033 (−0.058 to 0.007)		−.310	.16
Heel strike angle (°)	−.088 (−0.160 to −0.017)	−.305	.02	−.026 (−0.074 to 0.021)		−.141	.28
Stride length (m)	−.001 (−0.003 to −0.0005)	.174	.13	−.002 (−0.003 to −0.001)		−.349	.004
Distance (m)	−.662 (−1.464 to 0.140)	−.184	.10	−.786 (−1.321 to −0.252)		−.340	.005

^a^autoHSCT: autologous hematopoietic stem cell transplant.

^b^Linear regression models adjusted for age, sex, and BMI, with Benjamini-Hochberg false discovery rate adjustment set at α=.05, and all significant *P* values remained significant.

^c^ROM: range of motion.

## Discussion

### Principal Findings

To the best of our knowledge, this study is the first to measure pretransplant mobility in patients with hematologic cancer using an innovative system of wearable inertial sensors to characterize patients’ mobility compared with that of healthy adults and determine whether symptoms may identify patients with altered mobility characteristics. Mobility was significantly worse for patients than for controls, indicating that chemotherapy may directly or indirectly alter systems that control turning, gait, and balance. Among patients, those with high levels of nausea and pain before transplant had worse gait characteristics, demonstrating a conservative gait pattern of slow shuffled walking associated with functional limitations and fall risk [53-55].

### Comparison With Previous Studies

Wearable inertial sensors that measure multiple characteristics of turning, gait, and balance could better describe the mobility patterns affected by induction chemotherapy than self-report or field tests. Although other studies have only assessed gait, typically using a timed single walk test, our wearable sensor detected an aggregate of gait alterations in patients, along with differences in turning and balance. Gait parameters in our sample of patients were similar to those in a previous analysis using insole-worn sensors in a small sample of patients several months after allogenic HSCT [24]; however, our study provided great sensitivity by including additional gait metrics. These findings are consistent with slow and conservative gait patterns comparable with adults who are 20 years older [24,56], suggesting that patients may experience accelerated aging from induction chemotherapy [57]. The slow gait speed observed in our sample, consistent with previous findings in patients undergoing transplant [24], is concerning, given that slow gait speed at diagnosis is associated with subsequent hospitalizations and worse survival in older patients with hematologic malignancies [58]. Patients had a longer turn duration than controls, a measure associated with increased fall risk [59]; however, patients and controls took, on average, the same number of steps per turn. Increased double limb support time associated with falls [60] is a compensatory mechanism to make walking more secure with less time spent in single limb support. Gait may compensate for impaired balance [61,62], and thus, increased variability of gait characteristics could be owing to both compensatory mechanisms for balance deficits and multijoint incoordination. Control of balance while walking involves adjusting foot placement. Both variability in foot placement and double support time while walking also reflect impaired balance.

Postural sway during normal, quiet standing has long been shown to be a sensitive measure of balance control, with large, fast sway being associated with increased fall risk [63]. Consistent with other studies, primarily in survivors of breast cancer, patients exhibited worse balance, likely exacerbated by chemotherapy [26,64,65]. Sagittal and coronal sway values observed in our sample were worse than those previously associated with falls in elderly populations [66]. Chemotherapy can have neurologic and musculoskeletal impacts affecting mobility including distal sensory loss, ototoxicity, myelopathy, weakness, atrophy, and sarcopenia [67,68]. In addition, glucocorticoids coadministered during chemotherapy and deconditioning from hospitalization for cancer treatment lead to muscle loss that could also underpin decline in mobility [69,70]. These findings suggest that patients planning to receive autoHSCT may undergo a pretransplant mobility risk assessment to identify patients at the highest risk for falls and functional decline throughout their treatment trajectory. Moreover, pretransplant mobility assessment may allow clinical teams to prioritize limited rehabilitation expertise and resources for patients at the highest risk of functional decline and more extended hospital stays.

Symptoms may contribute to and co-occur with changes in mobility. Therefore, poorly controlled symptoms may help to identify patients at risk and those who may benefit from optimal symptom management and early palliative care integration [71]. We assessed multiple treatment-related symptoms previously associated with mobility and physical function in survivors of hematologic cancer [15-18]. In our sample, high nausea was significantly associated with great stride time variability and shallow heel strike angle. The pattern was also similar for pain, where high pain was significantly associated with slow gait speed, short stride length, great stride time variability, and less distance traveled. Persistent and severe nausea and pain clustering have been associated with poor performance status and limited physical function after cancer treatment [72]. Central nervous system disturbances owing to certain chemotherapies can affect cognition and movement, causing a sequela of symptoms comprising nausea and pain [73]. Chemotherapy can cause vestibular toxicity, resulting in nausea that intensifies over the transplant phase [74,75], which could directly or indirectly affect gait and balance [76]. Chronic pain is prevalent among survivors of hematologic cancer [77], has been associated with gait deficits in older adults [78], and is a significant risk factor for falls in survivors of cancer [79]. Persistent control of symptoms, including nausea and pain management, may be important for preserving physical functioning throughout the full treatment trajectory.

### Integration of Wearable Inertial Sensors in Clinical Care

Providers subjectively assess a patient’s functional status before autoHSCT using the Karnofsky Performance Status assessment—a tool with good reliability and validity, but which is subjective and prone to clinician bias [80-82]. Until recently, characterizing mobility was only possible with complex and expensive laboratory-based systems, making it difficult to assess patients at the point of care. Introduction of wearable inertial sensors to assess mobility in the clinic setting widens the scope of what can be learned and implemented in clinical practice [83]. Mobility Lab is an affordable (comparable with other mobile gait assessment platforms) and time-effective approach to assess patients for aspects associated with risk for functional decline including postural sway, spatial and temporal components of gait, and dynamic balance during common movement such as turning [36]. The average time for an in-clinic assessment is 15 minutes, and it provides clinically relevant and accurate mobility evaluation that could be integrated into patient care and inform clinical decision-making.

Detecting dynamic and potentially reversible gait changes during pretransplant appointments may minimize future health care use by directing resources to patients at high risk of treatment-associated disability or falls. Interventions to promote physical activity and exercise before or during treatment would improve physical function and mobility [84]. Exercise is feasible and can safely be initiated after induction chemotherapy [85]. Exercise interventions before autoHSCT have shown to improve quality of life and functional capacity, as measured by the 6MWT [86]. Symptom management itself may also lead to increased activity level in patients, and exercise has also been used to manage chemotherapy-related symptoms [87-89].

### Strengths and Limitations

A significant strength of our study was the use of wearable inertial sensors to obtain objective measures of mobility characteristics before autoHSCT. We were able to collect high-quality mobility data in the domains of turning, gait, and balance using wearable inertial sensors in a clinic setting, which may have future utility in patient care. Wearable inertial sensors have additional benefits including low cost and portability for assessment outside laboratory settings [90]. This study also has limitations. Our case-control analysis used a previously collected set of data on controls, causing the patient and control samples to be unbalanced on some characteristics such as sex and body composition (eg, height, weight, and BMI), which may influence mobility measures; thus, future studies should prospectively enroll a matched control cohort. Our sample size was modest for linear regression; thus, findings should be interpreted accordingly. We did not have access to data from previous induction chemotherapy (eg, chemotherapy drug or classification, dose, number of cycles, and weight change) and concurrent medication use (eg, antiemetics and pain medications); thus, we are limited in what can be inferred about symptoms assessed before transplant. Similarly, we did not collect data on physical activity levels, but it is possible that there may be interactions between symptoms, mobility, and physical activity. For example, patients with low symptom severity may be more physically active and therefore demonstrate better mobility. In contrast, patients experiencing nausea or pain may require increased need for rest, which negatively affects their mobility. Similarly, we did not have information about symptom management interventions that may have similar interactions. In addition, our cross-sectional analysis could not establish causality between gait characteristics and patient-reported chemotherapy-related symptoms. Thus, they may be co-occurring problems. However, it is possible that symptoms could serve as a surrogate indicator of developing mobility deficits. As the average time from induction chemotherapy to enrollment was approximately 3 weeks, patient-reported pretransplant symptoms may be related to chemotherapy or comorbidities. Future studies could better establish the temporality of symptom onset and progression regarding mobility using longitudinal serial assessment.

### Conclusions

Patients with hematologic cancer who have completed induction chemotherapy experience multiple alterations in mobility, as detected by a system of wearable inertial sensors. These altered gait patterns, which may have resulted from cancer treatment, place older patients with hematologic cancer at an elevated fall risk [91,92], which could ultimately increase morbidity and mortality risk [93,94]. Patients experiencing great nausea and pain at the time of autoHSCT may be at high risk of experiencing mobility limitations during and after transplant. Although this study could not infer whether chemotherapy-related symptoms directly alter gait, the findings highlight distinct mobility deficits in patients, which could not have been easily identified using standard mobility tests alone. Patients experiencing symptoms may warrant a more thorough assessment of their mobility using wearable sensors by the clinical team, including rehabilitation specialists, during routine appointments before hospitalization. Understanding these relationships could improve preventive care, symptom management, and rehabilitation efforts by identifying patients scheduled for autoHSCT who are at risk for further functional decline or falls after induction chemotherapy.

## Abbreviations

6MWT: 6-minute walk test
autoHSCT: autologous hematopoietic stem cell transplant
HSCT: hematopoietic stem cell transplant
MCID: minimally clinically important difference
REDCap: Research Electronic Data Capture

## Data Availability

The data that support the findings of this study are not publicly available owing to privacy or ethical reasons but may be provided from the corresponding author upon reasonable request. Restrictions apply to the availability of some data, which were used with permission of APDM, Inc for this study.
